# Delayed Diagnosis of Dirofilariasis and Complex Ocular Surgery, Russia

**DOI:** 10.3201/eid1902.121388

**Published:** 2013-02

**Authors:** Boris Ilyasov, Vladimir Kartashev, Nikolay Bastrikov, Rodrigo Morchón, Javier González-Miguel, Fernando Simón

**Affiliations:** Author affiliations: Rostov Oblast Diagnostic Centre, Rostov-na-Donu, Russia (B. Ilyasov);; Rostov State Medical University, Rostov-na-Donu (V. Kartashev);; Hospital of the North Caucasus Branch of the Russian Railway, Rostov-na-Donu (N. Bastrikov);; University of Salamanca, Salamanca, Spain (R. Morchón, J. González-Miguel, F. Simón)

**Keywords:** Dirofilaria repens, humans, retroocular nodule, optic nerve dislocation, ultrasound, parasite, differential diagnosis, optical, eye, ultrasonography, Doppler, MRI, magnetic resonance imaging, Dirofilaria

**To the Editor:** *Dirofilaria repens* is a vector-borne, zoonotic, filarial nematode that infects dogs, cats, and humans. In humans, *D. repens* worms cause subcutaneous dirofilariasis, characterized by the development of benign subcutaneous nodules that mimic skin carcinomas (*1*), and ocular dirofilariasis in orbital, eyelid, conjunctival, retroocular, and intraocular locations (*2*). Intraocular and retroocular dirofiliariasis causes considerable damage and discomfort in patients from the presence of the worms and from their surgical removal (*3*). Here, we report a retroocular *D. repens* nematode infection in a patient in Russia that illustrates the difficulties in clinical management and the inherent risks of surgical procedures to remove the worms.

A 20-year-old woman living in Rostov-na-Donu in southwestern Russia who had never traveled outside the city sought ophthalmologic consultation for pain and skin redness and swelling in the inner corner of the upper left eyelid. Swelling migrated successively to the temporal area, the lower eyelid, and the inner corner of the lower eyelid. The patient had no other ocular signs or symptoms, and her general condition was otherwise good. Results of ophthalmologic examination and routine laboratory tests were within normal limits. Four days of treatment with cefotaxime resulted in the remission of signs and symptoms. Approximately 2 months later, swelling in the inner corner of the upper eyelid appeared again, affecting the whole upper eyelid, without itching or tenderness. Allergies were diagnosed; cetirizine was administered for 4 days, and the signs remitted at the third day of treatment. One month later, marked upper left eyelid swelling occurred, resulting in ptosis. Cetirizine was prescribed again; edema subsided after 4 days of treatment but relapsed in the following 3–4 days. 

**Figure F1:**
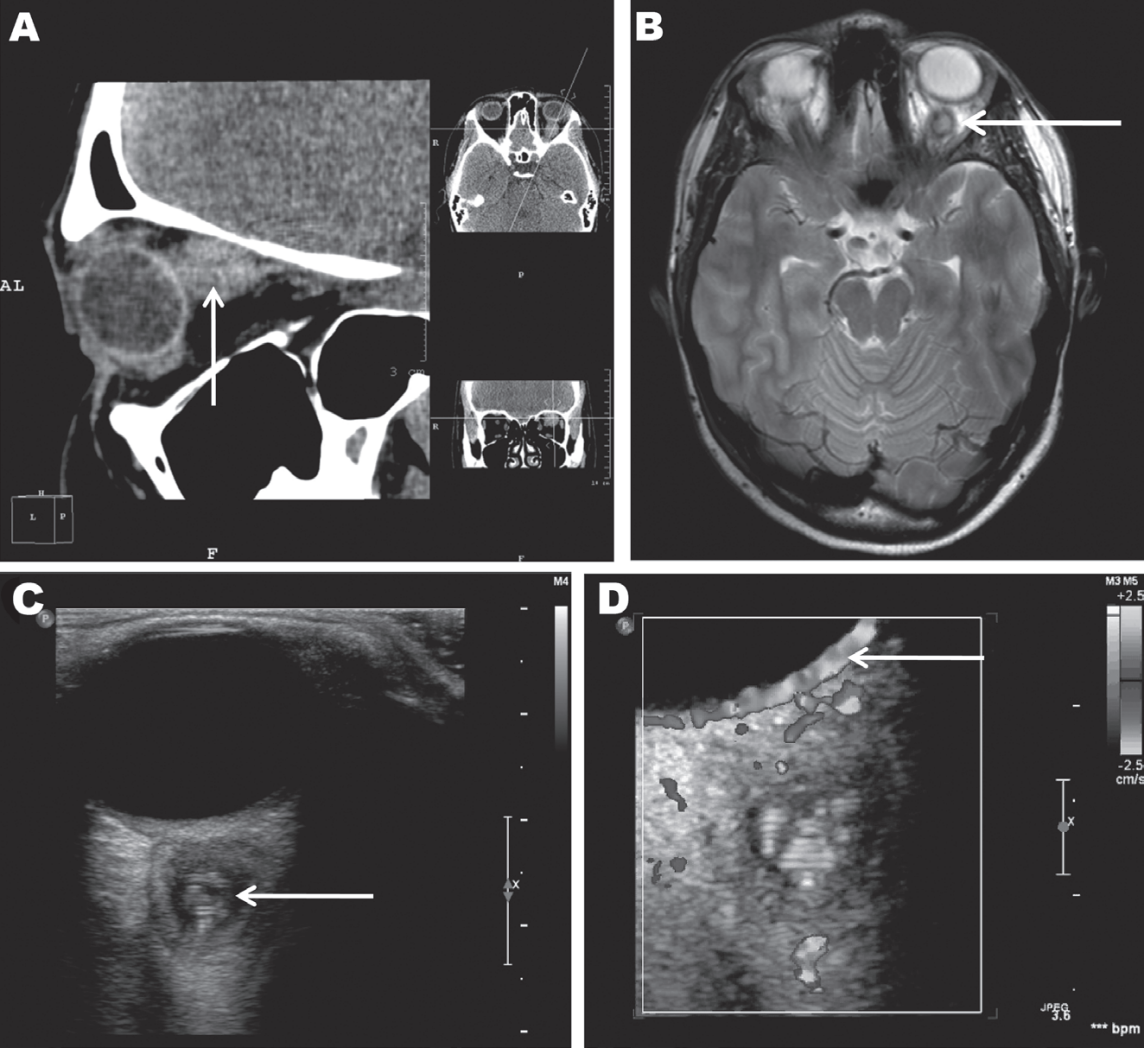
Retroocular nodule of a *Dirofilaria repens* worm detected in a 20-year-old woman, Rostov-na-Donu, Russia. The cyst (arrows) is shown by computed tomography scan (A) and magnetic resonance imaging (B). Ultrasonography images (C) show a worm-like structure inside the cyst (arrow), and color Doppler imaging (D) shows marginal vascularization of the lesion).

At least 4 subsequent relapses occurred; thus, a computed tomographic scan of the paranasal sinuses and orbits was performed, 4 months after signs and symptoms began (Figure, [Fig F1]panel A). The scan detected a soft tissue structure, 12 × 13 × 14 mm, behind the left eyeball, adjacent to and medially dislodging the optic nerve. No other abnormalities were found in the visible area of the brain and sinuses. Magnetic resonance imaging (MRI) performed 1 month later (Figure, panel B) corroborated the presence of a cyst-like structure with an irregular, rounded shape and clear, smooth borders closely adhered to the eyeball and optic nerve. T2-weighted images showed that the lesion had a high-density core but the surrounding tissue was low density. Adjacent to the lesion, the retrobulbar tissue was slightly swollen, the optic nerve was displaced medially and downward, and the adjacent upper muscle was displaced medially and upward. The diagnosis was evidence of a retroocular cystic lesion in the left orbit with a well-defined capsule and high-density but heterogeneous core structure. 

High-resolution ultrasound examination (Figure, panel C) revealed a well-defined, 3-mm, cyst-like wall containing fluid and dense, coiled-twisted linear internal structures that appeared to be actively moving (Video 1). Color Doppler examination (Figure, panel D; Video 2) revealed blood vessels in the wall but not inside the cystic structure. These additional examinations led to a diagnosis of a retroocular parasitic cyst in the left orbit, most likely a *Dirofilaria* spp. parasite. The parasitic cystic nodule was removed during a transpalpebral orbitotomy. A live, adult roundworm, 87 × 0.6 mm, was discharged from the cyst. Conventional PCR identified the roundworm as *D. repens* (data not shown).

**Video 1:**
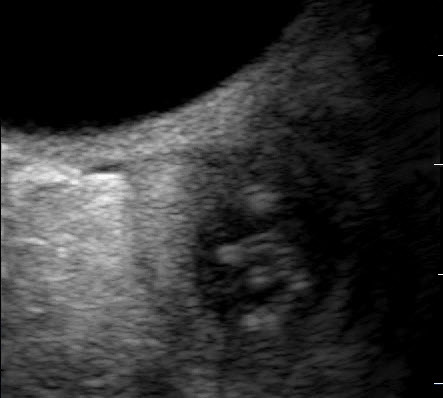
High-resolution ultrasound examination images showing a *Dirofilaria repens* worm actively moving inside retroocular nodule in a 20-year-old woman, Rostov-na-Donu, Russia.

Direct Video Link: http://streaming.cdc.gov/vod.php?id=eff01b6f9bbe41f1ea318db3d6e66f9220121218092846890

**Video 2:**
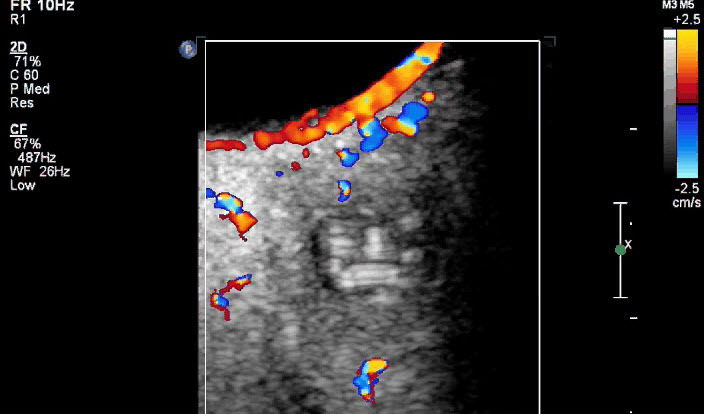
Color Doppler examination images showing the blood flow only outside the retroocular nodule in which the movement of a *Dirofilaria repens* worm is detected in a 20-year-old woman, Rostov-na-Donu, Russia.

Direct Video Link: http://streaming.cdc.gov/vod.php?id=e439085f91fad2cea4015df84bd7175220121219104320609

Although human subcutaneous *Dirofilaria* spp. nodules are benign, their detection may raise suspicion for a malignant tumor; thus, differential diagnosis is the key point in the management of human dirofilariasis (*4*). The case we described illustrates the difficulties of diagnosis when worms are in deep locations and patients experience unspecific and even unusual signs. These confounders resulted in a lengthy diagnostic procedure, with consequent detrimental physical and psychological effects on the patient. Even though the final diagnosis determined that the nodule was nonmalignant, its anatomic location required aggressive surgical intervention to remove it.

*D. repens* nematodes are spreading in Europe from the south toward the north and east (*5*–*7*) as a consequence of global warming, and prediction models have suggested incidence is increasing among animal and human hosts (*3*). Consequently, human ocular dirofilariasis will probably be found with increasing frequency in the future. Our experience illustrates that dirofilariasis should be included in the differential diagnosis of any nodule, independent of its anatomic location and the signs and symptoms shown by the patient. Moreover, ultrasonography represents a noninvasive technique that enables rapid preoperative identification of the parasitic origin of the nodules, thus avoiding unnecessary diagnostic delays. This technique is used for the diagnosis of cardiopulmonary dirofilariasis in animals (*8*) but has been used only sporadically for human dirofilariasis (*9*,*10*), which is habitually diagnosed postoperatively, after the surgical removal of the nodules or worms (*1*).
